# Refractory Status Epilepticus and Therapeutic Uncertainties: a Comprehensive Review on Targeting Neuroinflammation and Rationale for Developing a Platform Trial

**DOI:** 10.1002/advs.202509668

**Published:** 2025-09-29

**Authors:** Shanika Samarasekera, Suneesh Thilak, Asha Patel, Prashant Nasa, Zubair Ahmed, Lorraine Jacques, William J. Scotton, Cyril Chacko, Randeep Mullhi, Ruoling Chen, Vikram Patil, Suresh Renukappa, Tonny Veenith

**Affiliations:** ^1^ Department of Neurology University Hospitals Birmingham NHS Foundation Trust Birmingham West Midlands B15 2TH United Kingdom; ^2^ Department of Anesthesia Critical Care and Acute Care Medicine The Royal Wolverhampton NHS Trust Wolverhampton West Midlands WV10 0QP United Kingdom; ^3^ Faculty of Science and Engineering University of Wolverhampton Wolverhampton West Midlands WV1 1LY United Kingdom; ^4^ Neuroscience and Ophthalmology Department of Inflammation and Ageing School of Infection University of Birmingham Birmingham West Midlands B15 2TH United Kingdom; ^5^ Department of Neurosciences University Hospitals Birmingham NHS Foundation Trust Birmingham West Midlands B15 2TH United Kingdom; ^6^ Department of Neurology The Royal Wolverhampton NHS Trust New Cross Hospital Wolverhampton UK WV1 1LY United Kingdom; ^7^ Department of Critical Care University Hospitals Birmingham NHS Foundation Trust Birmingham West Midlands B15 2TH United Kingdom; ^8^ Faculty of Education Health and Wellbeing University of Wolverhampton Wolverhampton West Midlands WV1 1LY United Kingdom; ^9^ Department of Radiology JSS Medical College JSS AHER Mysuru Karnataka 570020 India; ^10^ JSS AHER‐ Wolverhampton Centre for Future Health and Policy Innovation Mysuru Karnataka 570020 India

**Keywords:** critical illness, epilepsy, immunomodulatory therapy, status epilepticus

## Abstract

Refractory Status Epilepticus (RSE) is a neurological emergency associated with considerable morbidity and mortality. The molecular mechanisms contributing to neuroinflammation in RSE are increasingly being recognized. Despite its severity, high‐quality and conclusive evidence is lacking for many RSE treatments, especially regarding the optimization of antiseizure medications and emerging immunotherapies. In this manuscript, the use of immunotherapy as a valuable treatment option in RSE is reviewed. The example of Toclizumab is used, its potential efficacy demonstrated by a case series from our center. Traditional clinical trial designs have proven inadequate in efficiently addressing these evidence gaps for this complex and heterogeneous condition. In examining the wider evidence for the use of anti‐inflammatory agents, including early immunotherapy, the scope for adaptive platform trials is explored to be utilized to develop an evidence base in this area. Neuroinflammation plays a role in propagating seizures and associated neuronal injury in RSE; these pathways may be amenable to immunomodulation. In this review, the limitations of existing observational data and the need for efficient, quickly translatable clinical trials are highlighted to evaluate multiple interventions for RSE. Innovative trial designs, such as adaptive platform trials, help generate robust evidence for rapid uptake in RSE.

## Graphical abstract

Refractory status epilepticus (RSE) is a life‐threatening neurological emergency with high rates of death and long‐term disability, but treatments for it are often based on limited evidence. RSE is now understood to involve neuroinflammation, which has led to the exploration of anti‐inflammatory and immunomodulatory agents like Tocilizumab. Traditional clinical trials are not well‐suited to study this complex condition, so innovative trial designs, such as adaptive platform trials, are urgently needed to efficiently evaluate new therapies and generate the high‐quality evidence required to improve patient outcomes.

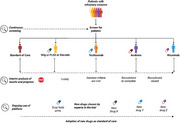

## 1 Introduction

Status epilepticus (SE) is a medical emergency characterized by continuous seizure activity or repetitive seizures without recovery of consciousness between episodes, lasting for 5 min or longer. When SE persists despite the administration of initial benzodiazepines and a suitable second‐line antiseizure medication (ASM), it is termed refractory status epilepticus (RSE).^[^ ^1^ ^]^ RSE poses a profound risk to life. In the United Kingdom, SE accounts for ≈2% of admissions to critical care units.^[^ ^1^, ^2^, ^3^ ^]^ The immediate mortality associated with SE is ≈15%, with a 1‐year mortality rate of up to 30% in adults,^[^ ^1^ ^]^ which rises dramatically as SE becomes more refractory. Indeed, mortality rates escalate from ≈15% in responsive SE cases to 25% in RSE and 40% in super‐refractory SE (SRSE), which is SE that continues or recurs despite 24 h of anesthetic therapy or re‐emerges during or after weaning from anesthesia. This suggests that a prolonged seizure results in a cascade of failed intrinsic inhibitory mechanisms, manifesting as reduced efficacy of ASM. SRSE, characterized by the failure of two distinct lines of therapeutic agents, signals a significant challenge in neurology.

A critical aspect of this progression is the dynamic change in neurotransmitter receptor populations. GABA‐A receptors, targets for benzodiazepines, for example, are progressively internalized during prolonged SE, leading to the pharmacoresistance of GABAergic agents over time.^[^ ^4^ ^]^ Prolonged and uncontrolled seizures lead to further excitotoxic neuronal injury, compounded by systemic and CNS effects such as hypoxia, hyperthermia, and acidosis, further exacerbating brain damage.^[^ ^1^ ^]^ Current treatment guidelines for status epilepticus (SE), such as those from the American Epilepsy Society, the Neurocritical Care Society, and the Intensive Care Society of the UK, provide a structured, stepwise approach to management.^[^ ^5^ ^]^ This typically involves rapid administration of benzodiazepines, followed by a second‐line drug therapy if seizures persist. The evidence base supporting treatments later in the pathway for more refractory stages of SE, including RSE and SRSE, is sparse and of lower quality.

The Established Status Epilepticus Treatment Trial (ESETT) provided crucial insights into second‐line therapies.^[^ ^5^ ^]^ The findings indicated no significant difference in efficacy among the three commonly used intravenous drugs. Each of these agents was effective in successfully terminating seizures and improving responsiveness in only 45–47% of cases. While ESETT suggested equipoise, it highlighted that half of these patients progressed to RSE despite the use of evidence‐based second‐line therapy, indicating limitations for existing therapeutic options for RSE. Many interventions employed for RSE and SRSE, including anesthetic agents (such as propofol, midazolam, barbiturates, ketamine) and immunotherapies (such as Anakinra, Tocilizumab, and Rituximab), are often administered based on data from anecdotal case reports, case series, observational studies with very limited numbers of patients, expert opinion, or extrapolation from their use in other conditions, and without robust level 1 evidence from randomized controlled trials (RCTs).

## 2 Neuroinflammation and Immunomodulation in RSE

Evidence suggests that neuroinflammation plays a role in the initiation, propagation, and perpetuation of RSE.^[^ ^6^ ^]^ This is particularly evident in conditions such as New‐Onset Refractory Status Epilepticus (NORSE), characterized by RSE occurring in a patient de novo, and its subtype, Febrile Infection‐Related Epilepsy Syndrome (FIRES), predominantly observed in the padiatric population.^[^ ^1^ ^]^ An underlying inflammatory or autoimmune process is often suspected or identified in these syndromes. Prolonged seizures with RSE and NORSE are associated with elevated serum and CSF levels of pro‐inflammatory cytokines, such as interleukin‐6 (IL‐6) and tumor necrosis factor‐alpha (TNF‐α).^[^ ^7^ ^]^ IL‐6 has been identified as a potential key mediator of inflammation in this context.^[^ ^8^ ^]^ Neuroinflammation disrupts the blood‐brain barrier, potentially accelerating the inflammatory cascade and contributing directly to neuronal damage. This link between inflammation and RSE suggests that a neuroanatomical substrate can be targeted to modify neuronal excitability and augment conventional ASMs.

The traditional understanding of SE pathophysiology centers on neuronal hyperexcitability as a critical mechanism in seizure generation.^[^ ^9^ ^]^ However, a growing body of research suggests a crucial role for neuroinflammation as an active facilitator of seizures, rather than an epiphenomenon.^[^ ^10^ ^]^ Prolonged seizure activity can lead to increased permeability of the blood‐brain barrier (BBB), allowing^[^ ^11^ ^]^ an infiltration of immune cells and inflammatory mediators into the central nervous system (CNS). Within the CNS, resident immune cells‐ primarily microglia and astrocytes, become activated and release a cascade of pro‐inflammatory cytokines (such as IL‐1β, IL‐6, and TNF‐α) and chemokines.^[^ ^12^ ^]^ These molecules enhance neuronal excitability by modulating ion channel function and neurotransmitter receptor expression, reducing the seizure threshold and promoting ongoing seizure activity. Sustained neuroinflammation contributes to excitotoxicity, oxidative stress and neuronal apoptosis and necrosis, exacerbating brain damage and contributing to long‐term epilepsy.^[^ ^13^ ^]^

This understanding provides a strong rationale for using anti‐inflammatory and immunomodulatory agents that directly target these cytokines in RSE. The use of agents such as Tocilizumab, an IL‐6 receptor antagonist, aligns with this mechanistic understanding.

## 3 Aims

This review aims to address the challenges of managing and developing an evidence base for RSE by:

1. Discussing an illustrative case series using Tocilizumab as an example of preliminary data highlighting the potential of immunomodulatory agents.
2. Summarizing pathophysiological rationale and observational clinical evidence supporting anti‐inflammatory and immunomodulatory therapies in RSE.
3. Proposing an integrated RSE clinical trial strategy incorporating immunomodulatory agents, guided by the latest international consensus guidelines from the Intensive Care Society.
4. Advocating the adoption of platform trials as an essential innovative strategy to accelerate the generation of high‐quality evidence for RSE therapies.

## 4 Current Approaches for Managing SE and RSE

The management of status epilepticus (SE) typically follows a stepwise approach, beginning with rapidly acting benzodiazepines and followed by second‐line intravenous ASMs such as levetiracetam, phenytoin (or fosphenytoin), or valproate if seizures persist. While this initial phase is well‐defined by guidelines and supported by evidence, such as the ESETT trial, the therapeutic options become less clear as SE progresses to RSE and SRSE.

In RSE and SRSE, treatment often involves inducing anesthesia using continuous infusions of anesthetic agents, including propofol, midazolam, barbiturates (e.g., pentobarbital, thiopentone), or ketamine.^[^ ^1^ ^]^ However, the optimization of these agents, including their dosing, duration of use and criteria for weaning, is guided mainly by expert opinion and limited research rather than high‐quality evidence. RSE management is complicated by the phenomenon of diminishing therapeutic returns with increasing seizure duration. The pharmacoresistence of ASMs, including benzodiazepines, significantly increases as the seizures continue.^[^ ^14^ ^]^ For instance, one retrospective study found that patients treated within the first 30 min of seizure activity experienced successful seizure termination with a reduction in mortality and neurocognitive morbidity, however, this response rate dropped further in patients with ongoing seizure activity.^[^ ^5^, ^15^ ^]^ This time‐dependent pharmacoresistence is attributed to pathophysiological changes within the seizing brain, such as the internalization of GABA‐A receptors, which reduces the effectiveness of GABAergic agents.^[^ ^16^, ^17^ ^]^ The failure to achieve rapid seizure control alters the brain's responsiveness to interventions, creating a vicious cycle of ongoing seizures, neuronal injury and neuroinflammation, with an exacerbation of pharmacoresistence. Escalating ASMs alone (especially those with similar mechanisms of action) may therefore be insufficient in RSE. Alternative neurotherapeutic strategies are also required.

The lack of definitive evidence for RSE and SRSE treatments is reflected in the considerable heterogeneity of our clinical practice. A survey of neurocritical care practitioners in the United States revealed that two‐thirds of institutions lacked a standardized protocol for treating NORSE.^[^ ^18^ ^]^ Although a broad consensus recommendation highlighting the potential role of immunotherapy for the treatment of both NORSE and FIRES was published in 2022.^[^ ^19^ ^]^ The timing and choice of immunotherapy remain at the discretion of the treating center. In the absence of an evidence‐based treatment approach, this variability can further contribute to patient mortality and morbidity, highlighting the urgent need for evidence‐based therapeutic strategies.

## 5 Illustrative Case Series of Tocilizumab for RSE in our center

Tocilizumab was used in five patients with RSE over a three‐year time interval. The cohort comprised patients with RSE of mixed etiology, both NORSE and RSE in the context of established epilepsy. Tocilizumab was administered in all but one case within 14 days of RSE onset. All patients had failed to respond to a minimum of three ASMs and adjuvant first‐line immunomodulatory treatment with intravenous methylprednisolone (IVMP) in all but one case–two where IVMP was not administered because of initial concern about concomitant infection. Second‐line immunomodulation with plasma exchange (PLEX) was used in three of the five cases. Baseline characteristics of the cohort are presented in **Tables** **1** and **2**. The primary outcome, termination of status epilepticus, was achieved within 72 h of Tocilizumab administration in all five patients. However, functional outcomes were poor: four patients had a Modified Rankin Scale (MRS) score of ≥ 4, indicating significant disability at the time of hospital discharge, and one patient died following a prolonged admission. Baseline functional status, multimorbidity, pre‐existing disabilities and immunocompromised states are likely to have contributed to these poor functional outcomes.

**Table 1:** Patient details included in the case series. ACV–acyclovir, B/L–bilateral, ASM–antiseizure medication, CBZ–carbamazepine, CLB–clobazam, CLN‐ clonazepam, CTR–ceftriaxone, DEX–dexamethasone, ICU–intensive care unit, GI–gastrointestinal, HIV–human immunodeficiency virus, HTN–hypertension, IVMP–intravenous methylprednisolone, LCM–lacosamide, LEV–levetiracetam, LZM, lorazepam, MDZ–midazolam, PHT–phenytoin, PPF–propofol, MRS–modified Rankin score, PLEX–plasma exchange, RSE–refractory status epilepticus, SE–status epilepticus, TAZ = piperacillin and tazobactam, VPA–valproic acid.

Case	Age, Sex	Suspected Etiology of RSE	Co‐Morbidities	MRI of the Brain on Admission	ASMs	Anesthetic Agents	Other Therapies Administered Before Tocilizumab	Day of Tocilizumab Administration	Resolution of SE Post Tocilizumab (days)	Length of Stay in ICU (days)	MRS on Discharge	Length of Hospital Stay (days)
**1**	19, F	Infective meningoencephalitis	Nil	Restricted diffusion involving B/L external capsules. Diffuse ependymal enhancement along the lateral ventricles, occipital region, and margins of brainstem.	CLN, LCM, LEV, LZM	MDZ, PPF	ACV, CTR, DEX, IVMP, PLEX	11	1	13	1	61
**2**	28, F	Hypoxic brain injury	Alcohol dependency, Anxiety, Depression, HIV	Diffusion restriction involving the deep grey nuclei and cerebral cortices, with corresponding FLAIR hyperintensity, consistent with global hypoxic ischaemia.	LCM, LEV, LZM	MDZ, PPF		9	1	27	5	67
**3**	49, M	Decompensation of epilepsy following upper GI bleed	Alcohol dependency, Focal epilepsy, Gastritis, HTN	Subcortical FLAIR hyperintensity in the right parietal region, multiple supra and infratentorial microhaemorrhages of the frontal and parietal lobes	CBZ, LCM, LEV, LZM, PHT	MDZ, PPF, thiopentone	ACV, CTR, IVMP,	5	3	29	Died	Died day 94
**4**	41, F	Immune‐mediated encephalitis (No antibody identified)	Alcoholic liver disease, Depression, and previous GI bleeding	FLAIR abnormality in B/L mesial temporal/hippocampal region. A signal change is seen in the subcortical frontal and parietal lobes **Post tocilizumab (day 133)**: near total resolution of previously identified changes	CBZ, CLB, LCM, LEV, LZM, PHT	MDZ, PPF	ACV, CTR, IVMP, PLEX,	71	1	17	4	157
**5**	20, M	Decompensation OF epilepsy following COVID‐19 infection and bacterial pneumonia	Focal epilepsy, Intellectual disability	Not performed CT Head reported as no structural abnormalities	CBZ, CLB, LCM, LEV, LZM, PHT, VPA	MDZ, PPF	CTR, co‐trimoxazole, DEX, TAZ	8	3	17	4	51

**Table 2:** Cerebrospinal fluid analysis. CSF biofire tests: Haemophilus influenzae, Listeria monocytogenes, Neisseria meningitidis, Streptococcus agalactiae, Streptococcus pneumoniae, Cytomegalovirus, Enterovirus, Herpes simplex virus 1, Herpes simplex virus 2, Human herpesvirus 6, Human paraechovirus, Varicella zoster virus, and Cryptococcus neoformans/Gatti.

Case		Polymorphs	Monocytes	RBC	Protein [g L^−1^]	Glucose[mmol L^−1^]	Negative Findings
1	Day 7	2	6	<1	0.22	3.8	CSF biofire. NMDA receptor, AMPA1/2 receptor, Anti‐DPPX, CASPR2, LGI1, and GABAB1/2 receptor
	Day 22	<1	<1	<1	0.19	5.1	CSF biofire. Cryptococcal antigen, TB culture, acid‐fast bacilli. NMDA receptor, AMPA1/2 receptor, Anti‐DPPX, CASPR2, LGI1, and GABAB1/2 receptor
2	Day 2	0	0	35	0.27	Not tested	CSF biofire. Toxoplasma DNA, TB culture, acid‐fast bacilli. Bilirubin and oxyhaemoglobin
	Day 7	< 1	< 1	8	0.18	Not tested	CSF biofire. TB PCR negative and acid‐fast bacilli. Cryptococcal antigen. Mycobacteria culture
3	Day 54	< 1	< 1	< 1	0.26	4.4	CSF biofire. Cysticercosis Antigen ELISA, acid‐fast bacilli, Toxoplasma PCR.
4	Day 15	0	0	102	Not tested	2.7	CSF cytology. HSV I, HSV II, Varicella Zoster, CMV, adenovirus, enterovirus, JC virus, BK virus, herpes virus 6, paraechovirus, and EBV. GABA B1, NMDA, AMPA1, AMPA2, LGII, and CASPR2 antibodies.
	Day 20	5	0	1310	0.26	5.3	CSF cytology. GABA B1, NMDA, AMPA1, AMPA2, LGII, and CASPR2 antibodies.
5	Day 14	< 1	< 1	<1	0.28	Not tested	CSF biofire. CSF cytology. Absent oligoclonal bands.

This retrospective case series is limited by its small sample size, single‐center setting, and the potential for confounding by co‐interventions. It nevertheless demonstrates a potential role for IL‐6 receptor blockade with Tocilizumab in achieving seizure termination in RSE. It also illustrates the complex, highly refractory patient populations encountered in clinical practice. Third, it underscores the limitations inherent in small observational studies. Observed seizure cessation, while encouraging, cannot be definitively attributed to Tocilizumab without a comparator group, and the translation of seizure termination to longer‐term functional outcomes remains unclear. Achieving seizure control, especially if delayed, may not be sufficient to ensure good neurological outcomes since significant neuronal injury may already have occurred.^[^ ^18^ ^]^ These poor neurocognitive outcomes have important implications for the design of future clinical trials in RSE, and emphasize the need for assessment of longer‐term function in addition to immediate seizure control.

Tocilizumab was a later‐line agent within this cohort. This contrasts with the consensus recommendations for NORSE, which advocate an earlier consideration of second‐line immunotherapies such as IL‐6 antagonists, preferably within seven days of RSE onset.^[^ ^19^ ^]^ This case series reinforces the need for larger, prospective, randomized controlled studies to rigorously evaluate Tocilizumab's efficacy and safety and determine its precise place in the RSE treatment algorithm.

## 6 Therapeutic Opportunities for Anti‐Inflammatory and Immunomodulatory Agents in RSE

Recognition of neuroinflammation has led to the exploration of various agents with anti‐inflammatory/immunomodulatory properties that target different pathways, reflecting the complexity of the inflammatory processes (summarized in **Table** **3**).

**Table 3:** Summary of Key Anti‐Inflammatory and Immunomodulatory Agents in Refractory Status Epilepticus.

Agent	Mechanism of Action	Key Evidence	Typical Dosing	Potential Adverse Events
**Corticosteroids**	Broad anti‐inflammatory, immunosuppressive; ↓ cytokine production, ↓ immune cell trafficking	First‐line immunotherapy in NORSE/FIRES; Case series; Expert opinion.	Methylprednisolone 1 g IV daily for 3‐5 days or 20‐30 mg kg^−1^ day^−1^	Hyperglycaemia, psychosis, infections, hypertension, and electrolyte imbalance
**Intravenous Immunoglobulins**	Pleiotropic immunomodulation: anti‐idiotypic antibodies, cytokine modulation, complement inhibition	First‐line immunotherapy in NORSE/FIRES, Case series; Expert opinion	2 g kg^−1^ total dose over 2–5 days	Headache, aseptic meningitis, renal toxicity (rare), thrombotic events, infusion reactions
**Plasma Exchange**	Removal of pathogenic autoantibodies and inflammatory mediators	Option in antibody‐mediated or suspected autoimmune RSE; Expert opinion	Typically, 5–7 exchanges over 10–14 days	Hypotension, coagulopathy (due to citrate), infections, and access‐related complications
**Anakinra**	IL‐1 receptor antagonist; blocks IL‐1RA and IL‐1β signaling	Second‐line for cryptogenic NORSE/FIRES, Case reports/series	Variable, e.g., 100 mg SC daily to higher IV doses	Injection site reactions, neutropenia, infections
**Tocilizumab**	IL‐6 receptor antagonist; blocks IL‐6 signaling	Second‐line for cryptogenic NORSE/FIRES; Expert opinion case reports / series; small RCT (RSE).	4–8 mg kg^−1^ IV (e.g., as a single dose or repeated monthly)	Infusion reactions, infections (esp. respiratory), neutropenia, elevated liver enzymes, dyslipidaemia, GI perforation (rare)
**Rituximab**	Anti‐CD20; B‐cell depletion, ↓ antibody production	Recommended for suspected/confirmed antibody‐mediated RSE expert opinion; Used in NMDAR encephalitis	e.g., 375 mg m^−^^2^ weekly x 4 doses or 1 g x 2 doses 2 weeks apart	Infusion reactions, infections, Progressive Multifocal Leukoencephalopathy (PML) (rare), hypogammaglobulinemia

**(A) Corticosteroids (e.g., methylprednisolone)**: Corticosteroids exert broad anti‐inflammatory and immunosuppressive effects by inhibiting the production of multiple pro‐inflammatory cytokines, reducing immune cell trafficking, and stabilizing cell membranes. They are widely used as a first‐line immunomodulatory treatment in RSE, particularly when an autoimmune or inflammatory etiology, such as NORSE/FIRES, is suspected.^[^ ^20^ ^]^ The patients in our illustrative cohort also received intravenous methylprednisolone before Tocilizumab. Evidence supporting their efficacy in RSE is derived from case series, expert opinion, and their established role in other neuroinflammatory conditions.^[^ ^21^ ^]^

**(B) Intravenous Immunoglobulin (IVIG)**: IVIG has pleiotropic immunomodulatory effects, including neutralization of pathogenic autoantibodies via anti‐idiotypic antibodies, modulation of cytokine networks, inhibition of complement activation, and interference with Fc receptor function on immune cells.^[^ ^22^, ^23^ ^]^ IVIG is frequently used as an alternative or adjunct to corticosteroids or as a first‐line immunotherapeutic management of NORSE/FIRES. Like corticosteroids, its use in RSE is supported only by case series and extrapolation from its proven efficacy in other autoimmune neurological disorders.^[^ ^24^, ^25^ ^]^

**(C) Plasma Exchange**: Plasma exchange aims to remove pathogenic autoantibodies, immune complexes, and other inflammatory mediators from circulation. Plasma exchange is considered an option, particularly in antibody‐mediated RSE or patients with a high suspicion of an autoimmune etiology. Its efficacy is most established in conditions with known pathogenic antibodies.^[^ ^18^, ^26^, ^27^, ^28^ ^]^ Its use may be limited to larger centers with a collaborative renal dialysis unit.

**(D) Anakinra (IL‐1 Receptor Antagonist)**: Anakinra is a recombinant form of the human IL‐1 receptor antagonist, which competitively inhibits the binding of IL‐1Rα and IL‐1β1 to the IL‐1 receptor, blocking the pro‐inflammatory signaling of this cytokine. There is emerging evidence supporting the use of Anakinra in NORSE/FIRES, with several case reports and series, particularly in padiatric FIRES, suggesting benefits in patients refractory to other treatments.^[^ ^29^, ^30^ ^]^

**(E) Tocilizumab (IL‐6 Receptor Antagonist)**: Tocilizumab is a humanised monoclonal antibody which binds to soluble and membrane‐bound IL‐6 receptors, inhibiting IL‐6‐mediated signaling. IL‐6 is a pleiotropic cytokine with significant pro‐inflammatory effects in the central nervous system (CNS) and has been implicated in seizure activity and neuroinflammation.^[^ ^31^ ^]^ The evidence for Tocilizumab, in addition to our case series, includes a case series by Jun et al. (2018) involving seven patients with NORSE, in whom Tocilizumab terminated SE in 6/7 patients with a median onset of action of 3 days, although two patients developed severe infections.^[^ ^32^ ^]^ Several other case reports describe favorable responses to Tocilizumab in NORSE or FIRES, even when administered late in the disease course.^[^ ^33^ ^]^ A small, single‐center RCT from Egypt (n = 50 RSE patients) comparing Tocilizumab plus standard care versus standard care alone noted a significantly greater reduction in the Modified Status Epilepticus Severity Score (mSTESS) and levels of inflammatory markers (IL‐6, TNF‐α, IL‐1β, NF‐kappaβ).^[^ ^34^ ^]^ While this RCT has limitations (small cohort, single center), it provides a higher level of evidence than case series alone and supports the potential efficacy of Tocilizumab. A systematic review also suggested the efficacy of Tocilizumab, although the evidence was of low quality (involving small studies) with a substantial risk of bias. The review concluded its potential role in anti‐GAD65 antibody‐related refractory epilepsy.^[^ ^35^ ^]^

**(F) Rituximab (Anti‐CD20 Monoclonal Antibody)**: Rituximab is a chimeric monoclonal antibody that targets the CD20 antigen on B‐lymphocytes, leading to B‐cell depletion and a subsequent reduction in antibody production. It is primarily used in RSE associated with confirmed or strongly suspected B‐cell‐mediated autoimmune encephalitis (e.g., NMDAR encephalitis).^[^ ^31^ ^]^ The guidelines of Wickstrom et al. recommend rituximab for patients with suspected antibody‐mediated disease. A recent cohort analysis by Jang et al. (2024) suggested that the combination of Rituximab and Tocilizumab was associated with better long‐term outcomes in cryptogenic NORSE.^[^ ^1^, ^18^ ^]^

A new generation of clinical trials is necessary to compare the efficacy and side effect profiles of these immunomodulatory agents, ideally conducted within a multi‐arm platform trial framework. This would provide the framework for optimizing immunomodulatory strategies for RSE.

## 7 The Challenges of Clinical Trials in RSE

The slow pace of therapeutic advancement in RSE is attributable primarily to the difficulties in conducting traditional RCTs. RSE is a relatively uncommon medical emergency within the broader spectrum of seizure disorders. Defining the onset and offset of RSE is subject to the interpretation of both electroencephalographic and clinical findings.^[^ ^36^ ^]^ Access to continuous EEG monitoring is often limited outside specialist neuroscience centers.

RSE is characterized by substantial etiological and clinical heterogeneity, which poses considerable challenges to the design and execution of RCTs. Patient recruitment for RSE trials is notoriously difficult. The acuity and severity of RSE demand rapid intervention in incapacitated patients, leaving little time for delayed screening and consent procedures associated with traditional trials. The relatively low incidence of RSE means that achieving adequate sample sizes for statistically robust studies requires multi‐centre, often international, collaboration, which is logistically complex and expensive. Since RSE can arise from a multitude of underlying causes, the heterogeneous etiology of seizures results in differential treatment responses, potentially diluting the effects of treatment in broadly inclusive trials. Finally, the conventional RCT poses ethical considerations when applied to this critically ill population. Running separate, sequential RCTs for each promising agent or combination of agents in RSE would be prohibitively slow and resource‐intensive, delaying the identification of effective therapies for decades. Innovative designs for clinical trials are urgently needed to address these limitations.

## 8 Adaptive Platform Trials

**Figure 1:**
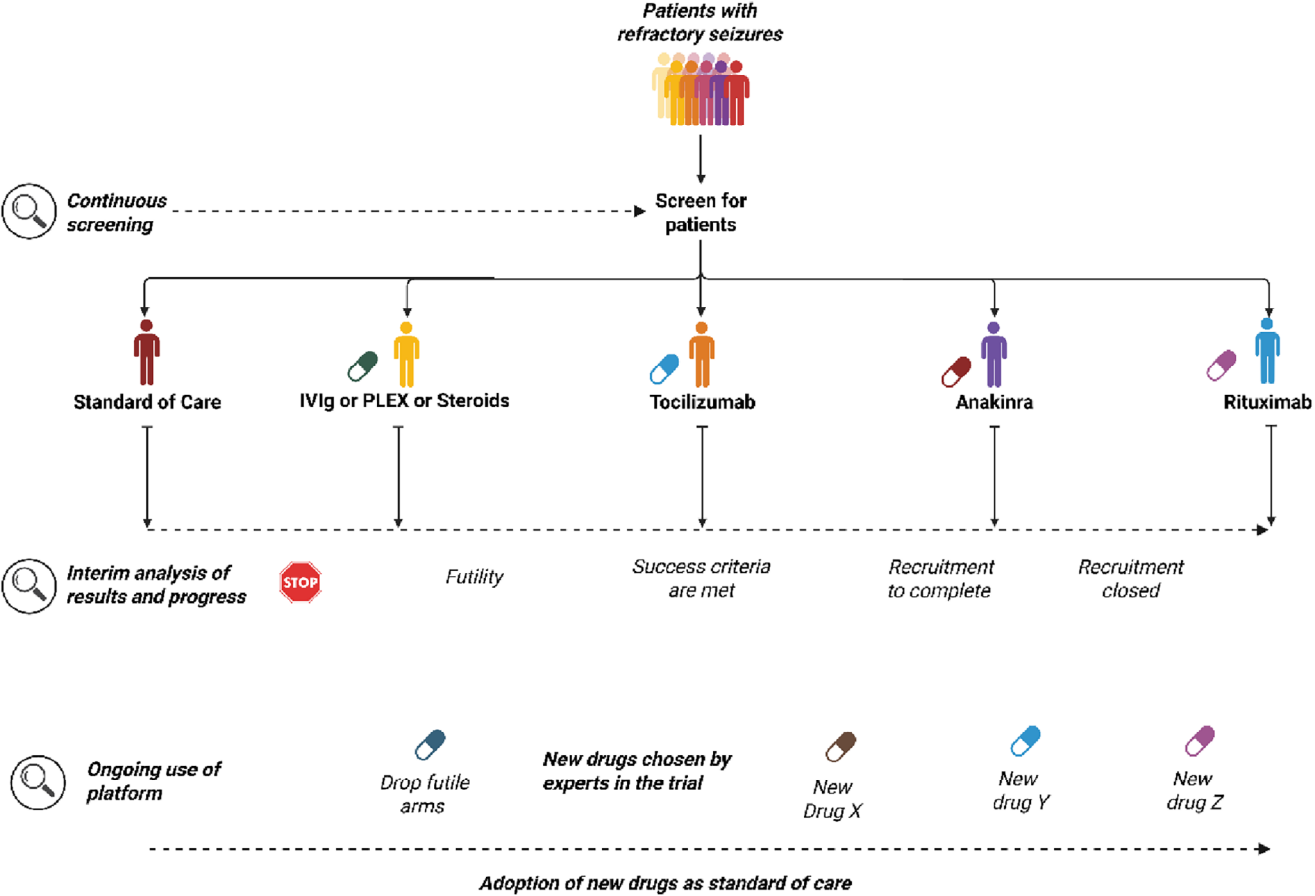
Example of a potential adaptive platform trial's schema for neuroinflammation in refractory status epilepticus (IVIg‐Intravenous Immunoglobulin, PLEX‐Plasma Exchange). Figure 1 was created with BioRender.com, released under a Creative Commons Attribution‐NonCommercial‐NoDerivs 4.0 International license(https://creativecommons.org/licences/by‐nc‐nd/4.0/deed.en).

Adaptive platform trials (APTs) represent a significant shift, enabling the continuous assessment of multiple interventions for a single disease or condition. Interventions can be added to or dropped from the platform based on pre‐specified rules outlined in a master protocol. Thus, a “standing trial” infrastructure (**Figure** **1**) allows for continuous learning and adaptation as new evidence emerges.^[^ ^37^, ^38^, ^39^ ^]^

Key features and advantages of APTs are:

**(A) Master Protocol**: A comprehensive protocol oversees the trial platform, including a common control group, standardized outcome measures, and consistent data management procedures. As a result, adding new therapeutic arms is streamlined, reducing the regulatory and ethical approval burden associated with each new intervention compared to starting separate trials.

**(B) Adaptive Randomisation**: Adaptive platform trials can employ response‐adaptive randomisation, where the probability of a patient being assigned to a particular treatment arm can be adjusted based on accumulating data on treatment efficacy and safety from within the trial. This can lead to a higher proportion of patients receiving more promising therapies.

**(C) Interim Analyses and Learning**: Regular interim analyses of accumulating data enable pre‐specified adaptations. These include stopping a treatment arm early due to overwhelming efficacy (success), futility (lack of benefit), or safety concerns. This “learn‐as‐you‐go” approach ensures that resources are focused on the most promising interventions and that patients are not exposed to ineffective or harmful treatments for longer than necessary.

**(D) Efficiency**: By sharing a common control group and infrastructure, APTs can evaluate multiple treatments more rapidly and with fewer patients than required for a series of independent, conventional two‐arm trials.

**(E) Ethical**: Patients are more likely to receive an active, potentially beneficial therapy. Ineffective or harmful interventions are identified and discontinued quickly, resulting in economic savings and sparing futile research efforts. This ability to adapt based on emerging data aligns APT research more closely with patient interests.

**(F) Flexibility and Sustainability**: Newer drug candidates can be added to the platform as they become available, allowing the trial to adapt rapidly to medical advancements and ensuring that the infrastructure remains sustainable and economically viable long term.

**(G) Subgroup Analysis**: APTs can be designed to explore treatment effects in pre‐defined patient subgroups, potentially facilitating the identification of tailored therapies.

Successful examples of APTs have emerged in various fields, including oncology, infectious diseases (notably during the COVID‐19 pandemic, with trials such as RECOVERY), and increasingly in neurology, with examples in stroke (e.g., STEP, ACT‐GLOBAL). The ESETT trial for established SE, although not a full platform trial, incorporated Bayesian adaptive randomisation with interim monitoring for futility and success, demonstrating the feasibility and benefits of adaptive methodologies that enable the calculation of probabilities in acute seizure disorders, comparing multiple active interventions. APTs are particularly well‐suited for conditions characterized by uncertainty, an urgent need for rapid knowledge generation, the availability of numerous novel therapeutic agents, and relatively short times to expected outcomes–all features that describe RSE.

## 9 Future Considerations

RSE remains one of the most challenging and life‐threatening emergencies in neurology, characterized by high rates of mortality and long‐term disability. Despite its severity, RSE management is hindered by a lack of high‐quality evidence for many therapeutic interventions. This review has highlighted the knowledge gap, limitations of current treatment strategies, and the urgent unmet need for more effective treatments.

The emerging understanding of neuroinflammation's role in the pathophysiology of RSE, especially in NORSE and FIRES, provides a clear rationale for exploring anti‐inflammatory and immunomodulatory therapies. Agents such as corticosteroids, IVIG, plasma exchange, Anakinra, Rituximab, and Tocilizumab are increasingly being used, with promising signals from case series, observational studies, and in the case of Tocilizumab, a small randomized trial. The illustrative case series on Tocilizumab, alongside other literature, suggests a potential benefit but also underscores the need for more definitive evidence. A structured approach, based predominantly on the 2025 international consensus recommendations by Mullihi et al.,^[^ ^1^ ^]^ offers a framework for integrating these immunomodulatory agents into RSE management, particularly advocating for their earlier consideration in appropriate patients. It is essential to recognise that this consensus‐based approach is built on a limited amount of direct comparative evidence for many of its components, particularly concerning the selection and sequencing of second‐line immunotherapies.

## 10 Limitations of this Review

The evidence base for many of the immunotherapies discussed in this review is still evolving. The information presented is predominantly derived from non‐randomized case series and expert opinion. This type of evidence has limitations, including the potential for recall and publication bias. While expert opinion can provide valuable clinical insight, it is limited by confirmation bias and, when considered in isolation, may not be generalizable. The proposed clinical trial strategy outlined in this review is based on the best available consensus and is therefore likely to change as new evidence emerges.

It's also important to note that small studies suggest long‐term functional outcomes from prolonged status epilepticus are often poor, with a third of patients experiencing neurological deficits and a mortality rate of 30% at 12 months.^[^ ^40^, ^41^ ^]^ This underscores the critical need for adopting innovative and efficient research methodologies, such as adaptive platform trials, to address the existing evidence gap in refractory status epilepticus. These trials can evaluate multiple interventions simultaneously, adjust based on accumulating data, and effectively identify successful therapies.

## 11 Conclusion

RSE is a neurological emergency that demands urgent improvements in both therapeutic strategies and the methods by which evidence for these strategies is generated. The traditional reliance on sequential, often underpowered trials has proven insufficient to address the complexities of RSE. Anti‐inflammatory and immunomodulatory therapies represent a significant avenue of promise as our understanding of the role of neuroinflammation in seizure perpetuation and neuronal injury has evolved. Preliminary findings, including our illustrative case series on Tocilizumab and recent cohort data examining the impact of both Rituximab and Tocilizumab on longer‐term function, highlight the potential role of these agents in managing RSE. A structured treatment approach should be adopted while acknowledging the pressing need for more robust evidence to support specific choices and sequences.

## Conflict of Interest

The authors declare no conflict of interest.

## Data Availability

The data that support the findings of this study are available from the corresponding author upon reasonable request.
